# Unilateral Atraumatic Femoral Neck Fracture in the Peripartum Period: Case Report and Literature Review

**DOI:** 10.7759/cureus.19524

**Published:** 2021-11-13

**Authors:** Artsiom Klimko, Alienor Brandt, Catalin Cirstoiu, Georgian Iacobescu

**Affiliations:** 1 Physiology and Neuroscience, University of Medicine and Pharmacy "Carol Davila", Bucharest, ROU; 2 Orthopedic Surgery, University of Medicine and Pharmacy "Carol Davila", Bucharest, ROU; 3 Orthopedics and Traumatology, University Emergency Hospital, Bucharest, ROU

**Keywords:** peripartum, trasient osteoporosis of the hip, regional osteoporosis, atraumatic hip fracture, pregnancy

## Abstract

Transient osteoporosis of the hip (TOH) refers to a temporary, focal reduction in bone mineral density that selectively affects bones in weight-bearing joints of young pregnant females. Due to inherent difficulties in diagnosing this pathology, it is difficult to estimate the incidence and it is conceivable that TOH is vastly underreported. In a rare subset of patients, TOH may progress to pathological fractures. We report a case of a 38-year-old pregnant woman who developed an atraumatic, displaced femoral neck fracture during her last trimester. Diagnosis and adequate management of TOH represents a clinical challenge as symptoms that precede the fracture are often non-specific, while the timing of the surgical treatment (i.e. before or after delivery) is debatable.

## Introduction

Complaints of pelvic pain during pregnancy are common and in a rare subset of patients this symptomatology may herald more severe pregnancy-related hip disease, such as transient regional osteoporosis [1]. As obstetricians are the primary care providers, identifying transient regional osteoporosis as an etiology of hip pain before it progresses to a fracture poses a significant clinical challenge. Excessive weight bearing load associated with rapid weight gain in pregnancy is a common benign cause of self-reported hip pain [2]. In its early stages, transient osteoporosis of the hip (TOH) has an identical presentation, manifesting as hip pain with insidious onset in the last trimester or early post-partum period - due to such difficulties associated with diagnosis, TOH is likely underdiagnosed. In rare cases, TOH progresses to transcervical, subchondral, or subcapital femoral pathological fractures, which serve as the inciting incidence for the eventual diagnosis of TOH.

## Case presentation

A 38-year-old Caucasian woman, 35 weeks into her first pregnancy, presented to the emergency department for acute right-sided hip pain which precluded weight-bearing. Her right leg was shortened and externally rotated - there was no bruising or evidence of trauma.

**Table 1:** Lab values upon admission. MCV: mean corpuscular volume; PT: prothrombin time; INR: international normalized ratio; aPTT: activate partial thromboplastin time

Lab value	Reference range	Lab value at admission
Complete blood count		
Hemoglobin (g/dL)	13-17.5	9.3
RBCs (x 10^6/uL)	3.6-4.9	2.97
WBCs (x 10^3/uL)	4-10	13.9
Hematocrit (%)	31-41	27.1
MCV (fL)	75-95	91.2
Platelets (x 10^3/uL)	179-408	225
Biochemistry		
Fibrinogen (mg/dL)	238-498	558
Blood glucose (mg/dL)	74-106	65
Creatinine (mg/dL)	0.51-0.95	0.35
Creatinine kinase-myocardial band (U/L)	0-24	18
Alanine aminotransferase (U/L)	5-35	31
Aspartate aminotransferase (U/L)	5-35	21
Coagulation Studies		
PT (seconds)	9.4-12.5	10.8
INR	0.8-1.1	0.98
PT (%)	80-130	102
aPTT (seconds)	22.0-36.0	32.0

**Figure 1:**
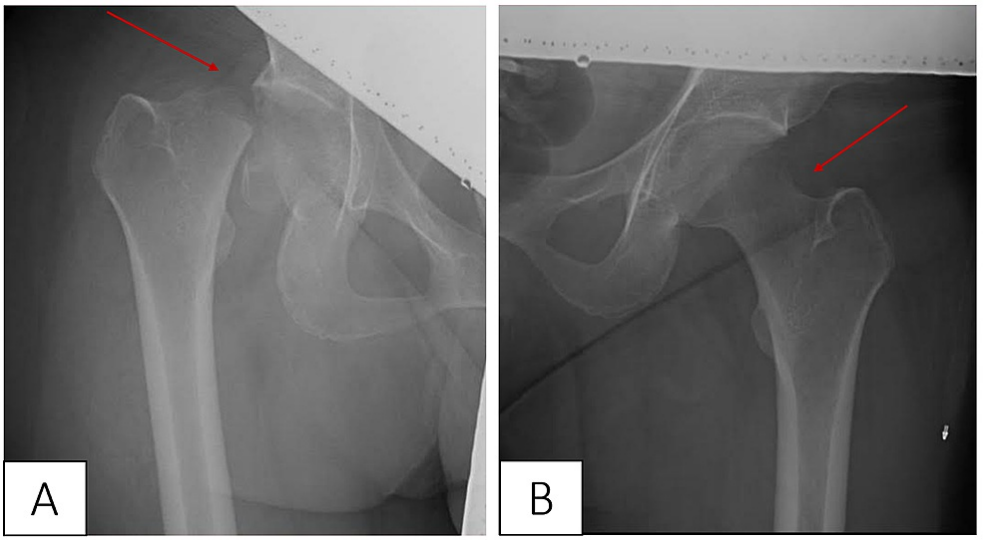
Hip radiography showing a right-sided displaced femoral neck fracture (A, red arrow) and normal lucency of the left hip (B, red arrow).

The patient’s history was significant for hereditary thrombophilia (Factor V Leiden) and secondary anemia. Hip radiography revealed an unstable, displaced, right-sided femoral neck fracture with no evidence of osteonecrosis (Figure 1). The decision to administer radiography, in this case, was based on the American College of Radiology guidelines, which cite an absence of in-utero deterministic effects of ionizing radiation effects after 27 weeks of gestation. Unfortunately, it was not possible to evaluate the symptoms of the patient with MRI at this time due to the coronavirus disease pandemic-induced stress on the healthcare system of our country.

The patient denied falls or trauma during the pregnancy, nor was there any history of smoking, alcohol abuse, use of glucocorticoids, or presence of rheumatologic/oncologic disease. Additionally, the patient was not malnourished, she underwent routine antenatal care, and took multivitamins. Serologic tests for inflammatory markers, as lab tests for serum calcium, phosphate, alkaline phosphatase, parathyroid hormone, vitamin D, and D-dimer returned normal.

**Figure 2:**
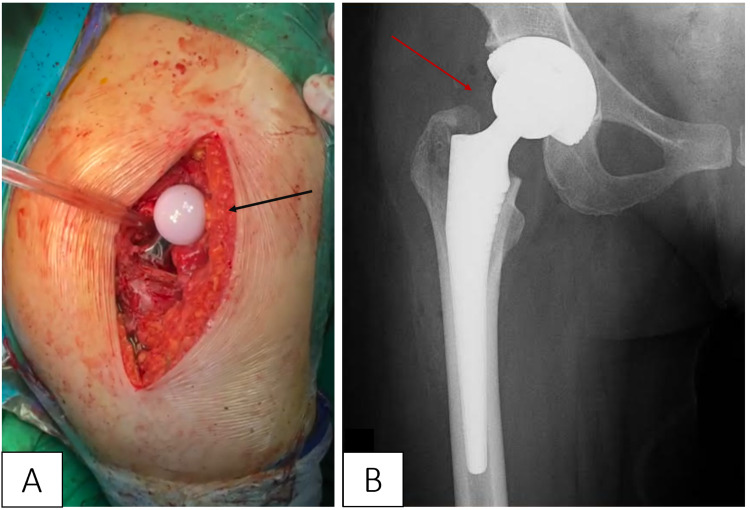
Installation of the total uncemented prosthesis intraoperatively (A, black arrow) and postoperative radiography confirming prosthesis placement (B, red arrow).

**Figure 3:**
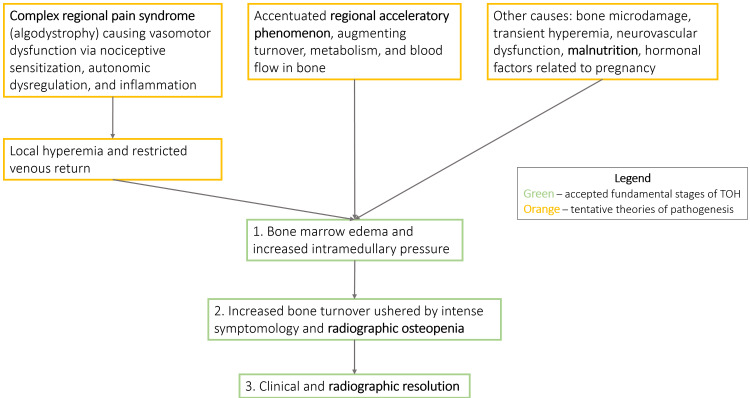
Summary of proposed pathogenesis of transient osteoporosis of the hip.

During multidisciplinary rounds, it was decided that delaying surgery was the best course of action out of fear of causing either mechanical or fluoroscopy-induced damage to the fetus during total hip arthroplasty. Five days later the patient experienced premature rupture of membranes, which was managed with emergency cesarean section (C-section) - no complications were encountered and a healthy 2300 g female was successfully delivered. Three days later the patient was transferred to our orthopedic surgery department for the treatment of the fracture. The significant degree of displacement (grade IV) of the fracture lasting over one week precluded open reduction with internal fixation due to fears of femoral head necrosis. During our literature review, we encountered a similar case of femoral neck fracture with grade IV displacement that was treated with open reduction internal fixation - despite restoration of blood flow to the femoral head within 15 hours, the authors still encountered femoral head necrosis with collapse six months later [3]. Given the considerable delay between symptom presentation and treatment, we decided the case warrants total hip arthroplasty instead of native hip salvage. Hemiarthroplasty was considered but was ultimately discarded as the conversion rate to total hip arthroplasty in young patients remain relatively high and the fracture was subsequently treated with a total uncemented prosthesis (Figure 2), consisting of a 50 mm cup with 32 mm ultra-high-molecular-weight polyethylene insert and a 32 mm head with a 4 mm ceramic insert (Link Inc., Hamburg, Germany). Postoperative radiography confirmed prosthesis placement (Figure 3); antibiotic and anticoagulant prophylaxis was initiated with ampicillin/sulbactam and enoxaparin sodium, respectively.

Three days after the intervention the patient developed moderate abdominal pain without fever and accelerated intestinal transit, which both worsened over the next four days. Given the clinical presentation, there was a high index of suspicion for infection with *Clostridium difficile* (*C. diff*), although the diagnosis was ambiguous as the enzyme immunoassay (EIA) for the *C. diff*-specific antigen glutamate dehydrogenase was positive, while EIA for exotoxin A and B were negative. Due to exacerbation of symptoms and development of moderate hypokalemia (2.9 mEq/L), empiric treatment with metronidazole was began and marked rapid improvement. The patient was discharged five days later.

## Discussion

In this case report, we presented a patient who developed a spontaneous, atraumatic femoral hip fracture, five days prior to delivery via cesarean at term. The most significant pregnancy-related hip disease includes entities such as TOH, osteonecrosis of the femoral head, and occult stress fractures. Osteoporosis refers to compromised bone strength - classically affecting postmenopausal women, where an abrupt decrease in estrogen augments bone resorption. In rare cases, the decrease in bone mineral density (BMD) is observed in atypical demographics, is transitory, and selectively affects bones of weight-bearing joints - TOH belongs to this group of multifactorial disorders, termed transient regional osteoporosis [1]. Prevailing theory separates these entities from pregnancy-associated osteoporosis, which is denoted by global skeletal BMD resorption, which when severe, usually manifests with thoracolumbar vertebral compression fractures [4]. TOH classically presents with sudden onset severe hip pain due to BME, which can predispose to femoral subchondral and neck fractures without notable trauma.

There is a paucity of definitive prevalence statistics for TOH occurring during pregnancy, as no systemic analyses exist due to the rarity of this condition. In a two-year prospective study, Steib-Furno et al. reported only three MRI-confirmed TOH cases out of 4900 pregnant female patients evaluated [5]. When the search was expanded to include all retrospective cases in the last 15 years, only 12 patients were identified to have a definite pregnancy-related hip disease: six patients had TOH (three cases were bilateral) - first symptoms appeared on average on the seventh month of parity, with a minor proclivity for the left side. The authors noted that only severe cases warranted referral and further workup at their department, thus creating a selection bias against mild cases. Confounding factors, such as atypical age of presentation or relatively common self-reported incidence of hip pain during pregnancy and the infeasibility to investigate such complaints with MRI likely contribute to notable underdetection.

In a retrospective case-control study of 33 TOH patients, Hadji et al. identified lack of physical exercise prior to puberty, dental problems in childhood, immobilization during pregnancy, and being overweight (body mass index > 26.0) as putative risk factors (our case did not have these risk factors) [6]. The study also reported a 12.1% incidence of hip fracture in the TOH group, as compared to matched controls. Pregnancy appears to be another major risk factor, as roughly one third of cases described in literature occurred either during pregnancy or in the immediate post-partum period, although the mechanism of TOH remains unclear [5]. TOH is a diagnosis of exclusion, which can be made after conditions like neoplasm, reflex sympathetic dystrophy, and avascular necrosis are ruled out. MRI is the most sensitive imaging modality to diagnose TOH. Low and high signal intensity in T1- and T2-weighted imaging, respectively, localize the osteopenic area, as well as aid in evaluation of BME.

The pathophysiology of TOH is still not completely understood - current theories postulate the progression to occur in three stages, summarized in Figure 4 [7-10]. With the advent of MRI, the observed BME became a cornerstone of the pathogenesis of the disorder, although the mechanism leading to this finding is still debated. Some implicate complex regional pain syndrome, formerly referred to as algodystrophy, and its sympathetic dysregulation-induced vasomotor dysfunction as the cause. Others implicate regional acceleratory phenomenon (RAP), which plays an instrumental role in rapid recovery following a fracture [7]. If RAP is to become pathologically prolonged, augmented turnover, metabolism, and blood flow in bone may explain BME [8]. In severe instances, BME may compress local vasculature and cause ischemic injury, thus drawing an exigent connection of TOH to osteonecrosis. Some consider TOH and osteonecrosis to be on the same pathological spectrum due to their clinical and radiographic similarities in the early stages.

TOH that progressed to an atraumatic unilateral fracture of the femoral neck was further explored through searching the PubMed and Scopus databases using the keywords “transient osteoporosis of the hip,” “displaced fracture,” “algodystrophy” to identify relevant case reports. The aforementioned keywords were identified within the title/abstract or as Medical Subject Headings (MeSH). A total of 56 case reports were identified, of which 46 were excluded due to being unrelated (i.e., bilateral fracture), published in a language other than English, or prior to 1990. A total of 10 cases were identified per our selection strategy and our findings are presented in Table 2.

**Table 2:** Summary of cases describing atraumatic unilateral fracture of the femoral neck due to transient osteoporosis of the hip. P-P: postpartum; TOH: transient osteoporosis of the hip; C-section: cesarean section; THA: total hip arthroplasty; ORIF: open reduction with internal fixation

Author and year	Patient age (years)	Gestation week of first symptoms and of the fracture	Gestation week of delivery and method	Suspected initial diagnosis	Type of fracture and side	Diagnosis confirmed with MRI	Treatment	Presumed underlying cause of TOH
Our case	38	35, 35	35, C-section	Hip fracture	Femoral neck, right side, displaced (grade IV)	No	THA	Idiopathic
Tayne et al., 2019 [10]	32	31, 38	38 weeks, C-section	Compressive femoral nerve neuropathy	Femoral neck, right side, displaced (grade IV)	Yes	THA	Hypoalbuminemia (1.6 g/dL) attributed to malnutrition
Kasahara et al., 2018 [11]	40	29, 3 days P-P	36, C-section	Excessive weight-bearing load	Subchondral, left side, collapsed femoral head	Yes	THA	Anorexia nervosa (BMI 15.4 kg/m2)
Guryel et al., 2010 [12]	31	38, 38	38, C-section	Hip fracture	Subcapital, left side, displaced (grade IV)	No	ORIF	Generalized osteopenia
Spinarelli et al., 2009 [13]	35	Last trimester, 2 weeks P-P	39, spontaneous vaginal	Low back pain syndrome associated with sciatica	Femoral neck, right side, partially displaced (grade III)	Yes	THA	Idiopathic
Cohen et al., 2007 [14]	37	19, 29	38, spontaneous vaginal	Hip fracture	Femoral neck, left side, partially displaced (grade III)	Yes	ORIF	Idiopathic
Wood et al., 2003 [15]	29	Last trimester, 5 months P-P	39, spontaneous vaginal	Hip fracture	Subcapital, right side, displaced (grade IV)	No	ORIF with muscle-pedicle bone graft	Idiopathic
Henry et al., 2003 [16]	24	38, 38	42, C-section	Sciatica	Femoral neck, right side, displaced (grade IV)	No	ORIF	Osteomalacia due to malabsorption syndrome
Fokter et al., 1997 [17]	20	23, 31	31, C-section	Sciatica	Subcapital, left side, partially displaced (grade III)	No	ORIF	Idiopathic
Junk et al., 1996 [2]	35	29, 2 weeks P-P	39, spontaneous vaginal	Excessive weight-bearing load	Subcapital, left side, displaced (grade IV)	No	ORIF with screws and plate	Idiopathic
Fingeroth et al. 1995 [18]	27	35, 35	39, spontaneous vaginal	Excessive weight-bearing load	Subcapital, right side, displaced (grade IV)	No	ORIF	Grand mal seizure

The majority of cases specifically described fracture of the femoral neck and as such, the severity was amenable to grading per the Gardner classification [19]. However, in some cases this approach was not applicable as the fracture involved either a subchondral head fracture or complete femoral head collapse [12]. The average age of the patients was 31.6 years (range 20-40). The classic clinical presentation included gradual onset groin pain with radiations to the buttock, greater trochanter, and anterior thigh - the pain was aggravated by weight-bearing and typically, there was no reduction in active or passive movement. In over half of cases, these symptoms were initially diagnosed as excessive weight-bearing load, compressive femoral nerve neuropathy, or sciatica. In only one case, the diagnosis was altered to TOH before the fracture occurred.

The average onset of these symptoms occurred on the 30th week of pregnancy and most patient were primiparous. If the fracture occurred during pregnancy, it did so on average on the 35th week of pregnancy. For the post-partum period, it typically ensued within the first two weeks. The fracture generally did not affect the extent of the surgery, except in one patient where the severe course of TOH, prompting C-section at 31 weeks of gestation. There does not appear to be a particular penchant of the fracture to involve a specific side (six right- and five left-sided) - seven patients presented with displaced, grade IV fractures. In most cases, the presumed underlying etiology of TOH malnutrition or idiopathic.

Guryel et al. noted that treatment of their grade IV displaced femoral neck fracture via open reduction with internal fixation resulted in avascular necrosis and femoral head collapse on six-month follow-up, despite restoration of blood flow to the femoral head in less than 15 hours after onset of symptoms [3]. Bone densitometry analysis in their patient also revealed T and Z scores of the lumbar vertebrae to be -2.26 and -2.24, which is in line with the study by Steib-Furno et al. where five of their six patients had moderately low lumbar BMD (although in their cohort, no patients experienced fractures) [5]. These findings confront convention by suggesting that TOH (and by extension, transient regional osteoporosis) may be a generalized state or at the very least, to co-exist with a generalized osteopenic state. More research is required to confirm this salient theory, as confirmation could potentially blur the distinction between this condition, generalized osteopenia, and pregnancy-associated osteoporosis.

Spinarelli et al. astutely diagnosed TOH before the appearance of the fracture [13]. BME was found to affect both hips - despite being prescribed antiresorptive therapy (alendronate and vitamin D) after delivery, the patient still presented with unilateral atraumatic femoral neck fracture 15 days after delivery. In another patient, where the likely catalyst of TOH was anorexia nervosa (BMI 15.4 kg/m2), the symptoms of the patient were exacerbated during five weeks of bed rest [12]. These two cases highlight a stern outlook regarding the utility of early diagnosis, as even avoidance of load bearing with antiresorptive therapy failed to prevent the advancement of TOH to a pathological fracture. Nonetheless conservative therapy with bed rest, analgesics, nonsteroidal anti-inflammatory drugs, oral or intravenous bisphosphonates, should be trialed with the aim of pain relief and prevention of microfracture progression [9]. In one case, it was unclear when the fracture occurred as the patient was wheelchair-bound for several weeks preceding the diagnosis; however, quadriceps and gastrocnemius muscle wasting was noted [16]. Additionally, the patient claimed the sciatica-like symptoms which heralded the femoral neck fracture, were also present during the prior two pregnancies.

Lastly, the fracture could have been caused by pathologic thrombosis secondary to Factor V Leiden. Atraumatic sacral insufficiency fractures caused by hypercoagulable states, such as antiphospholipid syndrome, have been reported [20]. However, other pathognomonic features include the history of miscarriages, deep venous thrombosis, pulmonary emboli -furthermore, management of antiphospholipid syndrome may involve immunosuppressive doses of glucocorticoids, which are known to cause avascular bone necrosis. None of those features were present in our patient and there are no reported cases of atraumatic bone fractures secondary to Factor V Leiden.

## Conclusions

Successful identification and management of femoral neck fractures in pregnancy hinges upon a comprehensive multidisciplinary approach. Sudden onset, debilitating hip pain should raise suspicion of TOH-induced fracture, especially if there are other signs of a hip fracture present on physical exam. In primiparous patients complaining of gradual onset hip pain, an MRI investigation may be warranted to exclude TOH - especially if such symptomatology was present during previous pregnancies in multiparous patients. In the case of TOH diagnosis, anti-resorptive therapy and bedrest may reduce microfracture progression and reduce the risk of contralateral hip fracture. Depending on the severity of the fracture, THA is a reasonable treatment modality, and surgery may be delayed to avoid mechanical or radiography-induced damage.
